# Gold nanoparticles as a substrate in bio-analytical near-infrared surface-enhanced Raman spectroscopy†
†Electronic supplementary information (ESI) available. See DOI: 10.1039/c4an01899k



**DOI:** 10.1039/c4an01899k

**Published:** 2015-03-24

**Authors:** Holly J. Butler, Simon W. Fogarty, Jemma G. Kerns, Pierre L. Martin-Hirsch, Nigel J. Fullwood, Francis L. Martin

**Affiliations:** a Centre for Biophotonics , Lancaster Environment Centre , Lancaster University , Bailrigg , Lancaster LA1 4YQ , UK . Email: f.martin@lancaster.ac.uk ; Tel: +44 (0)1524 510206; b Division of Biomedical and Life Sciences , Faculty of Health and Medicine , Lancaster University , UK . Email: n.fullwood@lancaster.ac.uk ; Tel: +44 (0)1524 593474; c Lancaster Medical School , Faculty of Health and Medicine , Lancaster University , UK

## Abstract

As biospectroscopy techniques continue to be developed for screening or diagnosis within a point-of-care setting, an important development for this field will be high-throughput optimization. For many of these techniques, it is therefore necessary to adapt and develop parameters to generate a robust yet simple approach delivering high-quality spectra from biological samples. Specifically, this is important for surface-enhanced Raman spectroscopy (SERS) wherein there are multiple variables that can be optimised to achieve an enhancement of the Raman signal from a sample. One hypothesis is that “large” diameter (>100 nm) gold nanoparticles provide a greater enhancement at near-infrared (NIR) and infrared (IR) wavelengths than those <100 nm in diameter. Herein, we examine this notion using examples in which SERS spectra were acquired from MCF-7 breast cancer cells incubated with 150 nm gold nanoparticles. It was found that 150 nm gold nanoparticles are an excellent material for NIR/IR SERS. Larger gold nanoparticles may better satisfy the theoretical restraints for SERS enhancement at NIR/IR wavelengths compared to smaller nanoparticles. Also, larger nanoparticles or their aggregates are more readily observed *via* optical microscopy (and especially electron microscopy) compared to smaller ones. This allows rapid and straightforward identification of target areas containing a high concentration of nanoparticles and facilitating SERS spectral acquisition. To some extent, these observations appear to extend to biofluids such as blood plasma or (especially) serum; SERS spectra of such biological samples often exhibit a low signal-to-noise ratio in the absence of nanoparticles. With protein-rich biofluids such as serum, a dramatic SERS effect can be observed; although this might facilitate improved spectral biomarker identification in the future, it may not always improve classification between control *vs.* cancer. Thus, use of “large” gold nanoparticles are a good starting point in order to derive informative NIR/IR SERS analysis of biological samples.

## Abstract 2

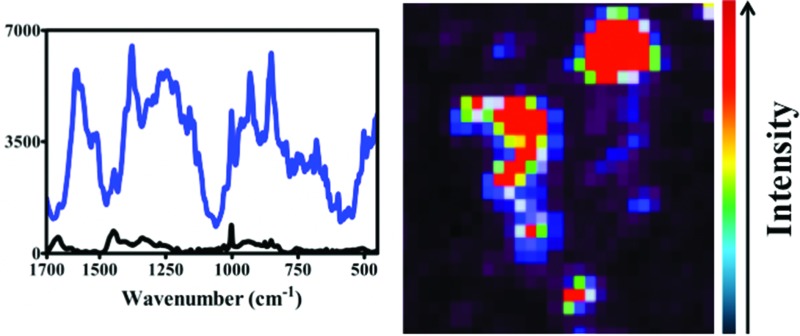
“Large” nanoparticles potentially are a good starting point in order to derive informative NIR/IR SERS analysis of biological samples.

## Introduction

Biospectroscopy techniques are gaining more widespread usage in the bio-analytical field due to their ability to interrogate samples across a wide range of biomolecules, providing detailed and specific (sub-)cellular information. The specific vibrational nature of chemical bonds facilitates the acquisition of spectra in the “biochemical fingerprint” region. Near-infrared (NIR) and infrared (IR) spectroscopies are beneficial for bioanalysis as biological molecules absorb radiation in these regions, unlike many non-biological samples.

Raman spectroscopy is a technique which has been employed extensively in the analysis of a variety of different biological samples,^1^ including different tissue types,^2^ individual cells,^3^ isolated cell components^4^ and biofluids.^5^ A key advantage of Raman over other IR spectroscopy techniques, such as Fourier-transform IR (FTIR), is the lack of interference from water. An absence of water interference is particularly advantageous for live-cell studies^6^ and for use *in vivo.* ^1^ Raman spectroscopy measures inelastic scattering caused by energy transfer between incident excitation photons and chemical bonds in a sample, which result in a change in the vibrational mode of the chemical bond and the energy, and thus the wavelength, of the scattered photon. This shift in wavelength is specific to particular molecular bonds, and readily interpreted from the output Raman spectrum.

However, there are significant limitations in the current usage of Raman scattering for biological purposes. It is much weaker than other scattering techniques, such as Rayleigh scattering or fluorescence, and thus biological samples which are typically weak Raman scatterers, may not give rise to an information-rich spectrum. The influence of fluorescence on Raman spectra is also problematic and can confound the biochemical signature; this influence can be reduced by using lasers at IR wavelengths. Additionally, cellular material is typically quite fragile and thus samples can be easily damaged by higher laser energies, introducing spectral artefacts into obtained data.

In order to overcome the limitations of conventional Raman scattering, it is possible to use surface-enhanced Raman spectroscopy (SERS). This is a phenomenon whereby the Raman signal of a target sample is greatly enhanced when placed into close proximity to a metal nanostructure.^7,8^ The nanoscale roughness necessary for SERS is present in many different types of metal nanostructures, including roughened electrodes, metal films and nanoparticles. In recent years, with the wide-scale production of metal nanoparticles, more novel forms of nanostructures have been identified as capable of generating a SERS effect. Nanostructure design for SERS experiments is important as the enhancement varies. The level of enhancement has been shown to reach up to 10^14^ times allowing the potential of SERS in single molecule detection.^9–12^

Nanoparticles potentially have a myriad of uses for SERS being able to specifically label sub-cellular regions both on the cell surface and within the intracellular environment.^13^ The dimensions of nanoparticles allow high localization of the SERS enhancement effect, permitting interrogation of a sample at the specific sub-cellular regions labelled.^14^ However, the degree of enhancement is dependent upon the physical parameters of the nanoparticles used and how they interact with the chosen excitation wavelength. Therefore, not all nanoparticle types will facilitate a large enhancement effect from the NIR or IR excitation wavelengths commonly used in bioanalysis. This means that optimization of the nanoparticle structure is required to gain sufficient enhancement from samples at these specific wavelengths. There are many influential factors including size, shape and composition that need to be considered for optimization of nanoparticle structure for different experimental Raman parameters;^15,16^ these have been elegantly represented previously.^17,18^

Optimal experimental parameters are dependent upon the sample, such as tissue type, individual cells or isolated cell components, *e.g.*, nuclei. Additionally, a particular analytical target, such as a specific protein target, may require specific labelling of metal nanoparticles to the target location, such as antibody binding.^19^ However, there are many samples with unknown targets for which the above labelling parameters are not relevant, *e.g.*, biofluids such as blood samples. Non-specific labelling of metal nanoparticles has been demonstrated using cationic gold labeling.^20^ Therefore, it is possible to use nanoparticles without any type of targeting molecules and to rely upon spontaneous associations of nanoparticles to biomolecules within/on the sample.

Many studies have used gold and silver nanoparticles that are 10 to 100 nm in diameter for SERS; however, theoretically these small gold and silver nanoparticles may not be optimal for use for NIR/IR SERS as their resonance wavelengths are within the visible or ultraviolet regions.^21^ It is important to consider that different metals have distinct responses under NIR/IR excitation. Gold and silver are good nanoparticle materials because they are unreactive and stable in solution compared to other metal nanoparticle types. Furthermore, they are easy to acquire, either commercially or through chemical preparation.^22,23^ There is a need to expand on SERS theory in order to find optimized metal nanostructures as SERS substrates for these excitation wavelengths. It is important to note that small nanoparticles have been shown experimentally to provide surface enhancement at IR wavelengths.^24^

An increase in the diameter of gold or silver nanostructures leads to a red shift in the resonance excitation wavelength, therefore moving the resonance wavelength towards the NIR/IR region.^21^ By increasing the size of the nanoparticles beyond the electrostatic approximation (typically a diameter >100 nm), more parameters become relevant, changing how the nanoparticle reacts with the incident excitation light.^25–27^ This has led to the theory that increasing the diameter of the metal nanoparticles used may be preferential for biological NIR SERS, thus increasing its potential as a novel diagnostic tool.

Routine point-of-care bioanalysis requires a simple but robust sample preparation procedure. In this study, we examine whether SERS using 150 nm *vs.* 40 nm gold nanoparticles could be applied robustly yet simply for bioanalysis. To this end, we examine if large gold nanoparticles (150 nm in diameter) give a strong SERS signal from MCF-7 cell samples. Secondly, we investigate the potential of non-specific labelling of nanoparticles (not attached to any targeting ligands) for the development of a strong SERS signal in samples without known or relevant targets for labelling, *e.g.*, biofluids. Such a protocol would be applicable for routine cancer screening or diagnostics.

## Experimental approach

### Gold nanoparticles

Gold nanoparticles [150 (designated “large”) and 40 nm (designated “small”)] were obtained from British Biocell International (UK) at a stock concentration containing 2.9 × 10^–4^ moles of gold per litre.

### MCF-7 cell analysis

**Fig. 1:**
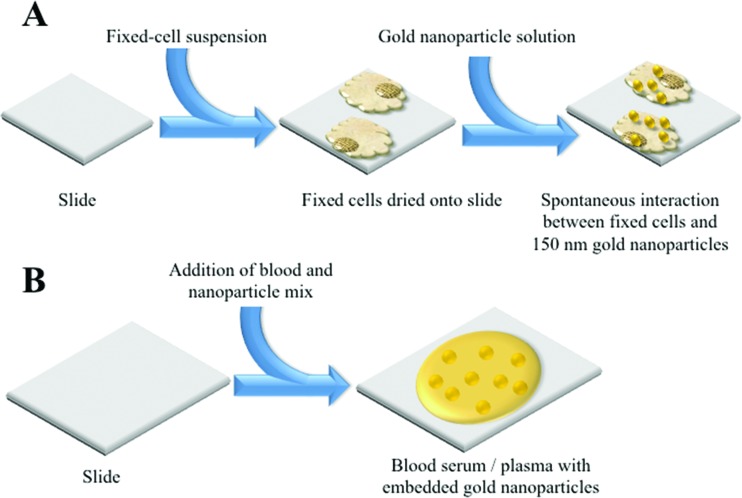
Schematic detailing the NIR SERS of sample preparations; (A) cellular sample; and, (B) biofluid sample.

MCF-7 cells were cultured in Dulbecco's Modified Eagle Medium (DMEM) (Lonza) with added foetal bovine serum (FBS) (Lonza) and penicillin/streptomycin mixture (10%). Cells were seeded in T25 flasks and cultured at 37 °C in 5% CO_2_ for 24 h. Once confluent, cells were disaggregated from each flask using trypsinisation. They were then fixed with 70% ethanol and 400 μl cell aliquots were placed on MIRR IR Low-E slides (Kevley Technologies, USA) and allowed to air-dry overnight. Nanoparticle solution (400 μl of 150 nm) was then applied to the dried cells and slides were again left to air-dry, before being placed in a desiccator (Fig. 1A).

**Fig. 2:**
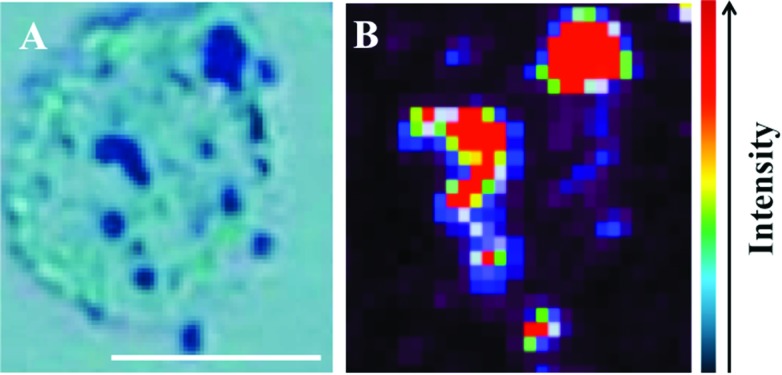
Fixed MCF-7 cells on MIRR IR Low-E glass slides with 150 nm gold nanoparticles subsequently added. A light micrograph image in (A) shows labelling of MCF-7 cell with 150 nm nanoparticles (dark regions), which co-localize with areas of high Raman signal intensity in (B) (red areas). Scale bar = 10 μm.

**Fig. 3:**
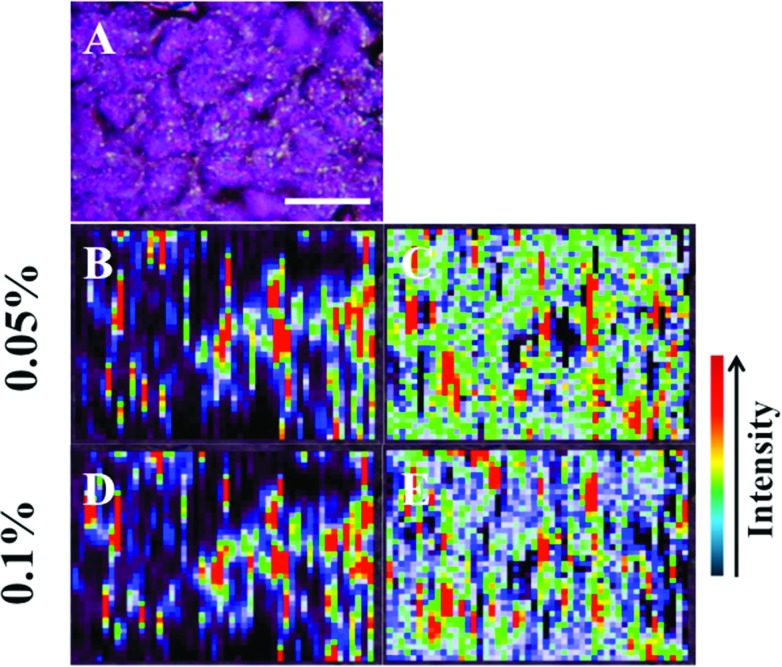
The presence of large nanoparticles allows analysis of large target areas rapidly using StreamLine™ Raman. A large cell clump seen by light microscopy (A) was analysed to quickly give false colour image maps; (B + D) = intensity at 1295 cm^–1^ (CH_2_ deformations); and, (C + E) = signal to baseline at 1194–1228 cm^–1^ (Amide III).^35^ Areas of high intensity (red) appear to correspond to areas of high nanoparticle localisation. Also, by increasing laser power, regions of relevant high SERS expression become easier to determine (B + C = 0.05% laser power; D + E = 0.1% laser power). Scale bar = 20 μm.

Raman spectra were acquired using an InVia Raman microscope (Renishaw plc, Gloucestershire, UK) equipped with a 100 mW 785 nm excitation laser, which was calibrated to 520.5 cm^–1^ using a silicon calibration source. Spectral maps of MCF-7 cells were acquired in a step-wise manner from the target area at 1 μm step sizes (Fig. 2). Spectra were acquired at 0.1% laser power at 50× magnification for 1 second and 1 accumulation. Analysis of an MCF-7 cell clump was acquired using StreamLine™ Raman analysis (Fig. 3) with an InVia Raman microscope equipped with a 150 mW 785 nm excitation laser, an exposure time of 10 seconds and 1 accumulation. Laser powers of 0.05% (0.075 mW) and 0.1% (0.15 mW) at source were used.

**Fig. 4:**
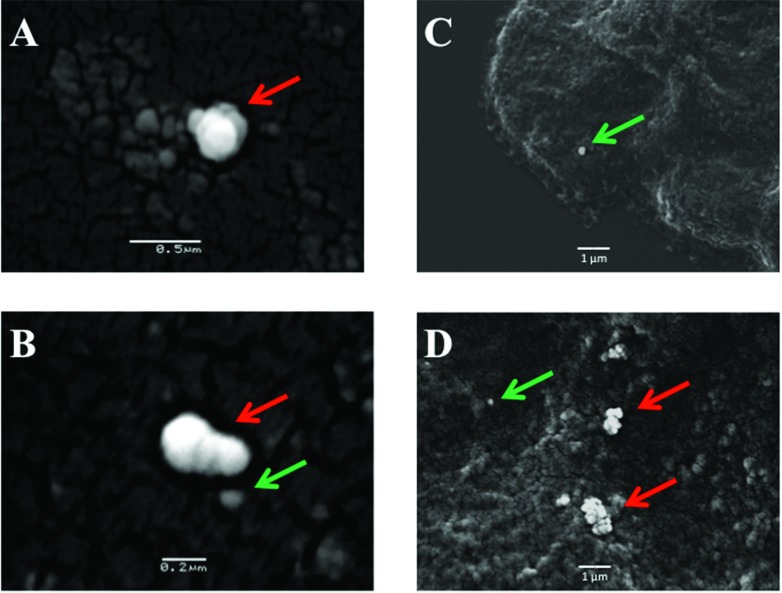
Scanning electron micrographs of the gold nanoparticles on the surface of the MCF-7 cells. (A) Shows an aggregated clump of 40 nm nanoparticles (red arrows); (B) shows an aggregated clump of 40 nm nanoparticles (red arrow) with a single 40 nm nanoparticle adjacent to it (green arrow); (C) shows a single 150 nm nanoparticle on the cell surface (green arrow); and, (D) shows at least two aggregated clumps of nanoparticles (red arrows) as well as a single isolated 150 nm nanoparticle (green arrow).

Post-SERS analysis, slides on which cells were deposited were processed for scanning electron microscopy (SEM) (Fig. 4). This involved mounting the slides onto aluminium stubs and gold-coating in a 150A Edwards sputter coater before examination at 15 KV in a JEOL 5600 digital scanning electron microscope.

### Blood plasma and serum analysis

**Fig. 5:**
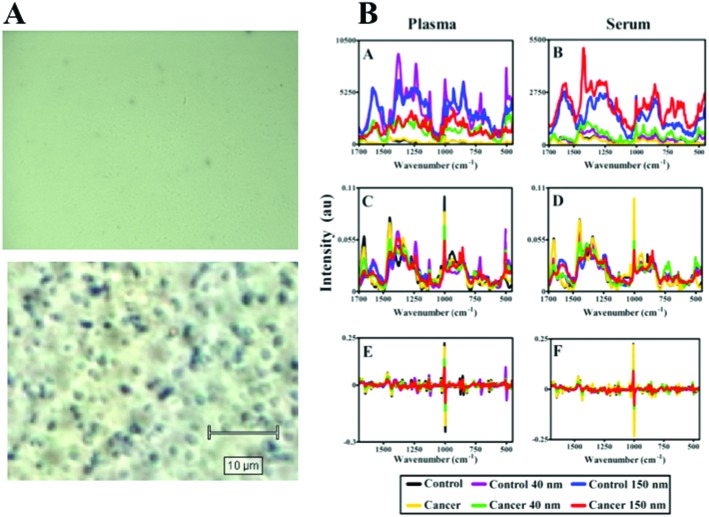
Influence of nanoparticles on SERS effect in blood plasma or serum samples. (A) Optical brightfield microscopy images of blood plasma samples with or without large (150 nm) gold nanoparticles. (B) Raman spectra (class means) of blood plasma (A, C, E) or serum samples (B, D, F) with or without gold nanoparticles following polynomial baseline correction to show raw enhancement (A, B), polynomial baseline correction followed by vector normalisation (C, D) and 1^st^ order differentiation followed by vector normalisation (E, F).

Samples were obtained from the Genitourinary Tissue Biobank at Lancashire Teaching Hospitals NHS Foundation Trust (Preston, UK) with ethical approval [Research and Ethics Committee (REC) approval no.: 10/H0308/75]. From age-matched cohorts of patients (*n* = 5 endometrial cancer, *n* = 5 non-cancer control), plasma and serum samples were taken from storage at –80 °C and thawed in a water bath at 37 °C for approximately 1 h. In order to compare the enhancement effect of nanoparticles at two distinct sizes, 200 μl aliquots of blood plasma or serum were mixed with 200 μl of stock 150 nm or 40 nm gold nanoparticle solution (Fig. 1B). The resultant mixture (total volume 400 μl) was applied to MIRR IR Low-E slides and left to air-dry. Control slides without nanoparticles were also prepared using 200 μl of blood plasma or serum sample and allowed to air-dry. Blood SERS spectra were taken at 10% laser power (2.4 mW at sample) at 50× magnification across the 500–2000 cm^–1^ spectral range for 10 seconds and 1 accumulation; a minimum of 25 spectra per sample slide were acquired. These air-dried samples could be examined under optical brightfield microscopy to demonstrate the presence or absence of nanoparticles (Fig. 5A). For transmission electron microscopy (TEM), gold nanoparticles (40 or 150 nm) were mixed 50 : 50 with blood serum and then 10 μl were pipetted onto carbon-/formvar-coated electron microscope grids (Agar Scientific, UK), blotted and allowed to dry before examination with a 10-10 JEOL TEM.

**Fig. 6:**
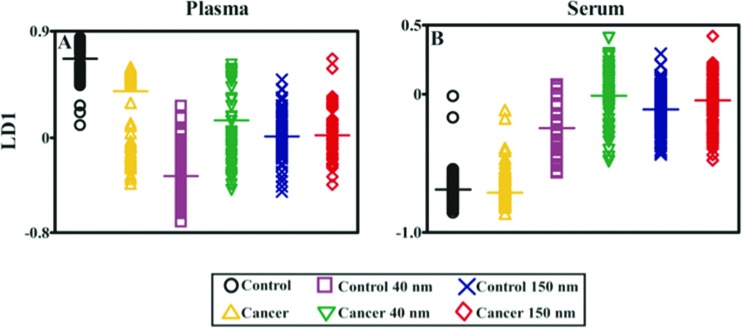
Classification of control *vs.* cancer Raman spectra following principal component analysis-linear discriminant analysis (PCA-LDA). Following 1^st^ order differentiation followed by vector normalisation, each spectrum is reduced to a single point in a PCA-LDA scores plot. For each class, the horizontal line represents the class mean.

Computational analysis was performed using MATLAB (Mathworks, Natick, USA) with an in-house developed toolkit (https://code.google.com/p/irootlab/), unless stated otherwise.^28^ The resultant Raman spectra were cut to 450–1700 cm^–1^ wavenumbers inclusive of spectral peaks present in the sample and wavelet de-noised. In order to display raw spectral enhancement, spectra were polynomial baseline corrected maintaining Raman intensity units (counts) (Fig. 5B-A, and B-B). For computational analysis, spectra were pre-processed using 1^st^ order differentiation followed by vector normalisation. Cross-validated principal component analysis (PCA) with optimised principal components (PC) factors followed by linear discriminant analysis (LDA) was conducted in order to discriminate between cancer *vs.* non-cancer patients (Fig. 6). Graphs were generated in GraphPad Prism 4.0 software (GraphPad Software Inc, CA, USA) and one-way analysis of variance (ANOVA) with Bonferroni post-hoc tests was conducted to determine *P*-values for separation between cancer *vs.* non-cancer (Table 1).

**Table 1:** Classification (control *vs.* cancer) of blood plasma or serum samples with or without SERS^*a*^

Class comparison	*P*-value	Significant
Plasma		
Control *vs.* cancer	<0.001	Yes
Control *vs.* cancer (40 nm)	<0.001	Yes
Control *vs.* cancer (150 nm)	>0.05	No
Serum		
Control *vs.* cancer	>0.05	No
Control *vs.* cancer (40 nm)	<0.001	Yes
Control *vs.* cancer (150 nm)	>0.05	No

^*a*^Following acquisition of Raman spectra, one-way analysis of variance (ANOVA) with Bonferroni *post-hoc* tests were conducted on class means per sample to determine *P*-values for separation between cancer *vs.* non-cancer.

## Results and discussion

The potential for 150 nm gold nanoparticles to generate good SERS enhancement is demonstrated; these larger nanoparticles allow for ready visualisation using optical microscopy (Fig. 2A). Fig. 2 shows an isolated MCF-7 cell labelled with 150 nm nanoparticles, which clearly demonstrates that regions of high Raman signal co-localize with the presence of the nanoparticles. Also, in Fig. 2B the signal appears to be highly localized to the regions surrounding the nanoparticles rather than being spread across the whole of the cell surface, supporting theoretical explanations of the SERS effect. As the cells were fixed prior to nanoparticles being added, one would expect that they would be adhered to the outer cell surface rather than having penetrated into the intracellular environment; therefore, the enhancement will be predominantly from the cell membrane nearest the nanoparticles. Post-SERS analysis using SEM (Fig. 4) shows that this is clearly the case. The nanoparticles adhere to the surface either as single entities or in aggregates. Compared to the smaller (40 nm) nanoparticles (Fig. 4A and B), the larger (150 nm) nanoparticles are much more readily detectable.

In Fig. 3, the application of rapid Raman scanning is tested on similar MCF-7 cell samples to those analysed in Fig. 2. Here, due to the capability of the StreamLine™ system to rapidly scan across a sample, a large clump of cells with 150 nm nanoparticle coverage was chosen for analysis. In the light microscope image of the sample (Fig. 3A), aggregates of nanoparticles this time appear as white spots across the cell surfaces. In false-colour image maps (Fig. 3B and D), areas of high nanoparticle expression show enhancement of the Raman signal. Also, Fig. 3C and E show that the enhancement is not just an increase in background signal but that relevant biological Raman signatures are present, calculated from the high signal-to-baseline intensity. These images show that, even despite the limiting factors of very rapid acquisition time and low laser power, enhanced biological spectra can be generated from large samples quickly using “large” nanoparticles. This allows for the potential of rapid SERS analysis of large tissue sections for diagnostics. Tissue sections parallel to conventional H&E staining may be mapped using SERS to facilitate high-throughput diagnosis.

The target area was analysed at two different laser powers, 0.05% (0.075 mW) and 0.1% (0.15 mW) in order to assess the sample with different laser exposures. It is more desirable to have very low laser powers to demonstrate the effectiveness of the SERS enhancement process. Previous studies have used similar laser powers to generate large signal enhancements from SERS samples.^19,20^ As the laser power is increased, it appears that areas of SERS expression became more evident with greater spatial resolution, and can still be clearly defined from those without SERS enhancement. Another advantage of large nanoparticles is that they can be observed optically (Fig. 2A, 3A and 5A), where they appear as black or white dots. The ability to see small aggregates of nanoparticles or individual nanoparticles allows areas where they are abundant to be manually targeted for analysis. This leads to highly-enhanced spectra being acquired more easily from a sample.

**Fig. 7:**
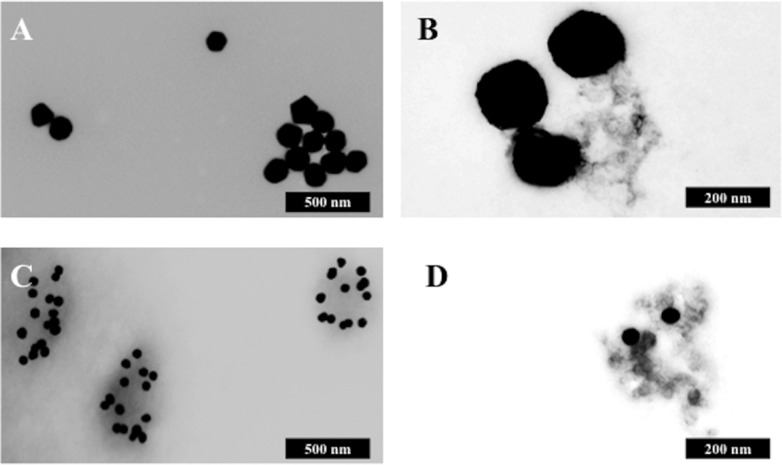
Transmission electron micrographs of the gold nanoparticles following mixing with blood serum. (A) Shows 150 nm nanoparticles mixed with serum; (B) shows 40 nm nanoparticles mixed with serum; (C) shows 150 nm nanoparticles mixed with serum; and, (D) shows 40 nm mixed with serum. Scale bar = 500 nm (A + C) or 200 nm (B + D).

Fig. 5B shows the analysis of blood plasma or serum samples with or without SERS in order to investigate its potential to differentiate between control *vs.* endometrial cancer samples. The search for blood-based cancer biomarkers is a very important area for bioanalysis and a novel use for biospectroscopy. Previous studies have investigated the possibility of biospectroscopy as a blood-based diagnostic tool.^29–31^ Fig. 5B-A and B-B show that either 40 nm or 150 nm nanoparticles generate a SERS effect, with the larger nanoparticles giving rise to the more pronounced enhancement in the protein-rich serum biofluid. Marked variation in the level of SERS effect even in the biofluids tested was noted (see ESI Fig. S1–S3†). As one would expect, when these spectra are normalised the SERS effect is less apparent (Fig. 5B-C and B-D); however, surprisingly many of the main peak intensities are higher in control compared to cancer. The ready observation of a SERS effect in such biofluids lends promise towards deriving and identifying novel spectrochemical biomarkers. However, the immediate objective of biospectroscopy is likely to be towards classification and diagnosis/screening of disease. To facilitate this, the Raman spectra were pre-processed using 1^st^ order differentiation followed by vector normalization prior to classification using PCA-LDA. Interestingly here, the use of smaller nanoparticles appears to give the best classification in both blood plasma and serum whereas the application of larger nanoparticles resulted in no between-class significance (Fig. 6). One explanation could be that aggregation of nanoparticles, even smaller ones, in a biofluid may be sufficient to give rise to an optimal SERS effect. Following TEM of 150 nm nanoparticles post-mixing with serum, it is noted that they form clusters, dimers and singlets (Fig. 7A). In this instance, the 150 nm nanoparticles in the clusters are in contact with each other and there is some variation in their shape; one is clearly pentagonal rather than spherical. In the case of 40 nm nanoparticles after mixing with serum, it is also observed that they form clusters with what are probably protein clumps (Fig. 7C). There are instances of the nanoparticles being in small groups of two to four, which are in contact. After mixing with serum, TEM shows 150 nm nanoparticles associated with what are probably serum proteins (Fig. 7B). Likewise, TEM shows 40 nm nanoparticles after mixing with serum; again, they appear to be associated with what are probably proteins (Fig. 7D).

An important point to make for the use of nanoparticles for larger studies, such as those for diagnostic development of NIR-SERS, is that the preparation process can be made incredibly rapid. Coupled with the rapid acquisition of SERS spectra, it is possible to quickly analyse multiple samples to potentially high sensitivity rates. The preparation is simple, allowing it to be utilized without specialized expertise. Whilst nanoparticles are suitable substrates, they do have some limitations for biological NIR-SERS. They are not very amenable to live-cell imaging due to the difficulty of cells to endocytose large nanoparticles through simple incubation.^32^ Approaches such as electroporation may facilitate this but this may lead to artefacts affecting any resultant spectra, distorting their reflection of underlying cellular biochemical structure. Through investigating differing nanostructures,^33,34^ other sensitive NIR or IR SERS nanostructures can be elucidated for use in bioanalytical research.^35^ Gold nanoparticles appear to be an optimal substrate for use in NIR or IR SERS. Ready enhancement of Raman spectra coupled with the rapid sample preparation and analysis increase the utility of large nanoparticles for biological NIR-SERS. This methodology greatly enhances the applicability of SERS as a high-throughput technology for disease diagnosis.
