# Supplementary material for: Gold nanoparticles as a substrate in bio-analytical near-infrared surface-enhanced Raman spectroscopy
Source: Analyst. 2015 Mar 24;140(9):3090–7. doi: 10.1039/c4an01899k (PMC4414298; doi:10.1039/c4an01899k)
Supplement: Supplementary file 1 [file AN-140-C4AN01899K-s001.pdf]

Electronic Supplementary Material (ESI) for Analyst.
This journal is © The Royal Society of Chemistry 2015

Electronic Supporting Information

Gold nanoparticles as a substrate in bio-analytical near-infrared surface-enhanced Raman scattering

Holly J. Butler[1] , Simon W. Fogarty[1,2], Jemma G. Kerns[3] , Pierre L. Martin-Hirsch[1], Nigel J. Fullwood[2,], Francis L. Martin[1,*]*
[1]Centre for Biophotonics, Lancaster Environment Centre, Lancaster University, Bailrigg, Lancaster LA1 4YQ, UK; [2]Division of Biomedical and Life Sciences, Faculty of Health and Medicine, Lancaster University, UK; [3] Lancaster Medical School, Faculty of Health and Medicine, Lancaster University, UK

Corresponding authors: Dr Nigel J. Fullwood / Prof Francis L. Martin PhD; *Email:* n.fullwood@lancaster.ac.uk / f.martin@lancaster.ac.uk; *Tel.:* +44(0)1524 593474 / +44 (0)1524 510206

Summary

Number of Pages: 4
Number of Figures: 3
Number of Tables: 0

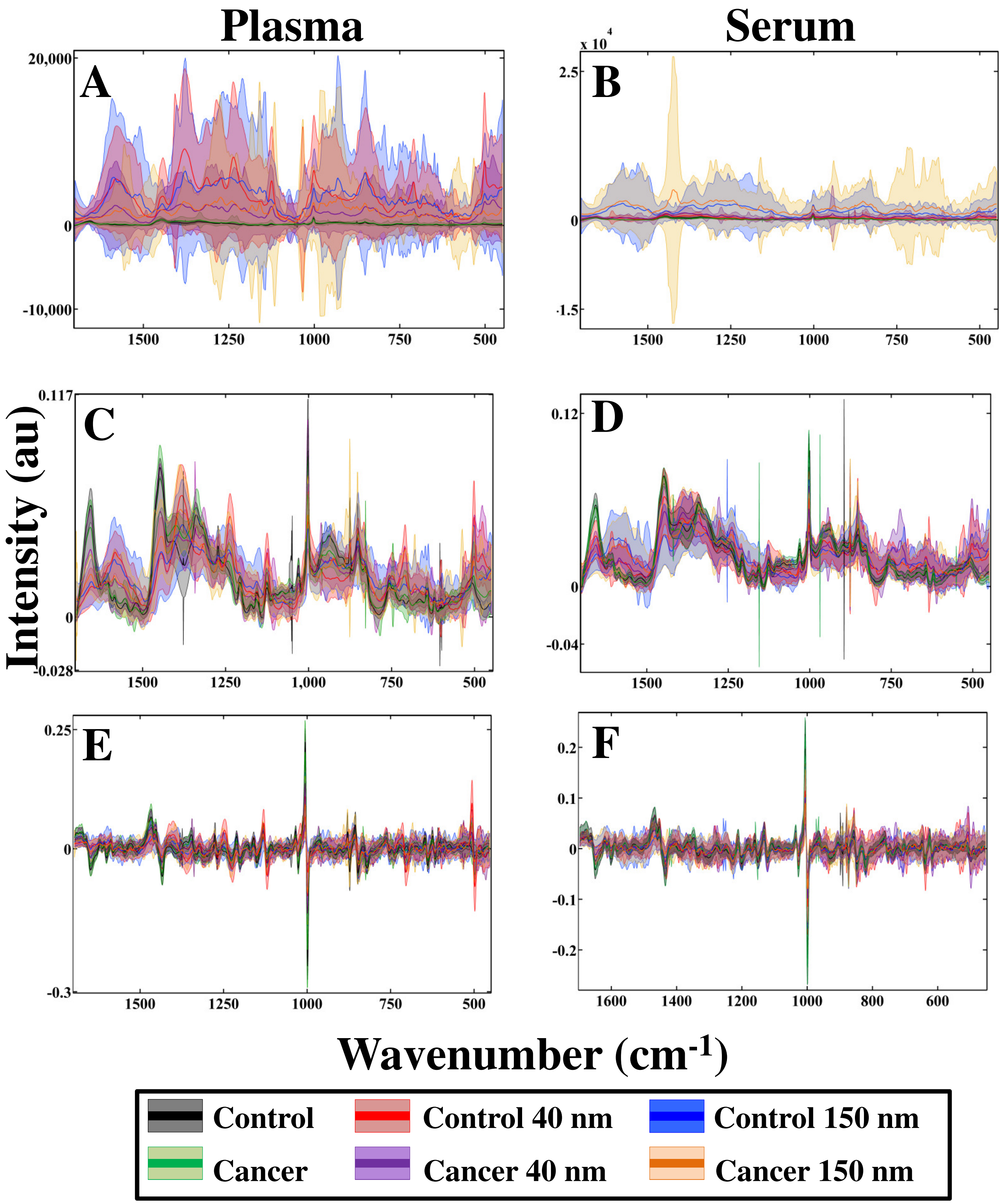

Figure S1. Influence of nanoparticles on SERS effect in blood plasma or serum samples. Raman spectra (class means with standard deviation) of blood plasma (**A**, **C**, **E**) or serum samples (**B**, **D**, **F**) with or without gold nanoparticles: following polynomial baseline correction to show raw enhancement (**A**, **B**), polynomial baseline correction followed by vector normalisation (**C**, **D**) or 1st order differentiation followed by vector normalisation (**E**, **F**).

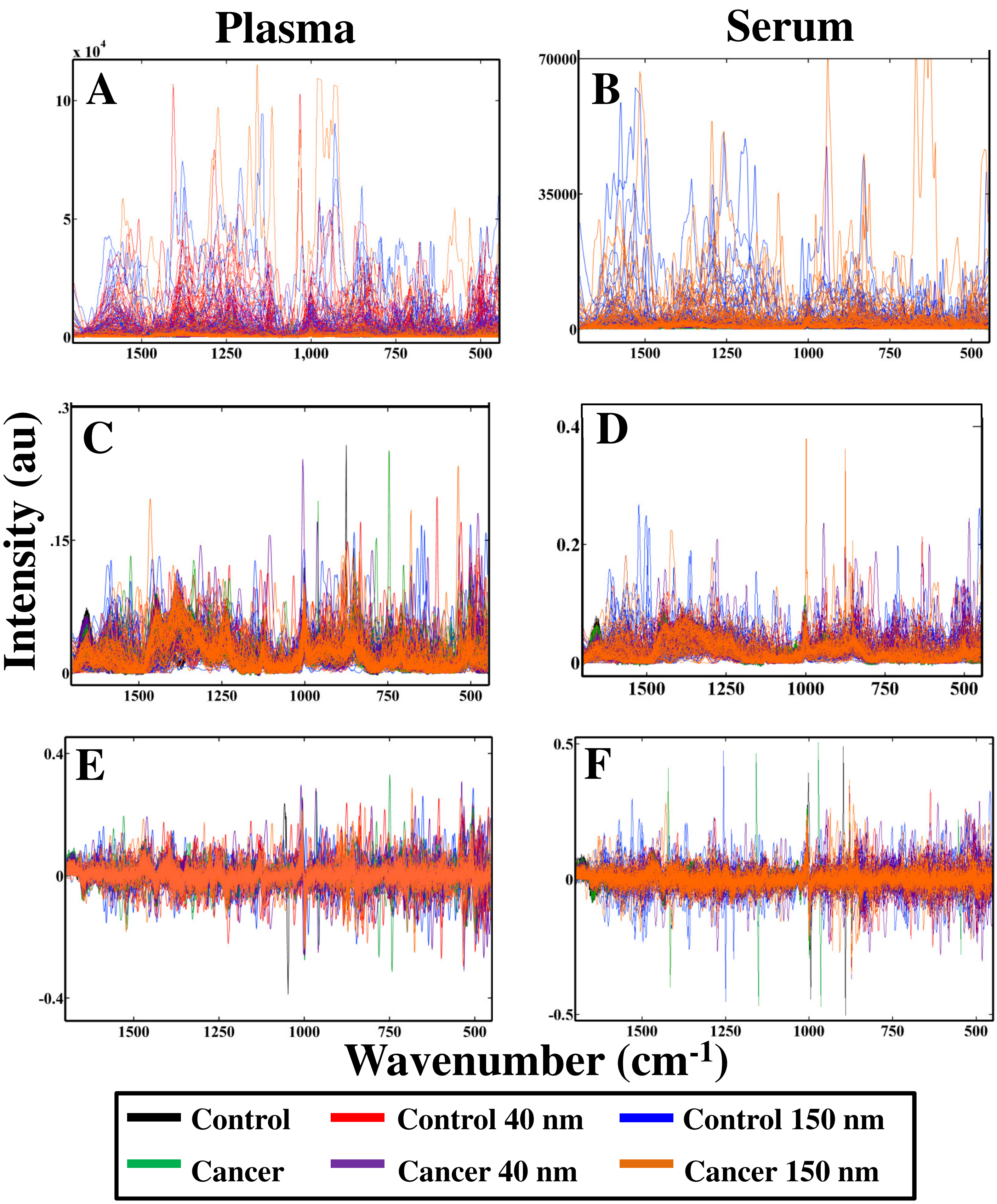

Figure S2. Influence of nanoparticles on SERS effect in blood plasma or serum samples. Raman spectra (all spectra in dataset) of blood plasma (**A**, **C**, **E**) or serum samples (**B**, **D**, **F**) with or without gold nanoparticles: following polynomial baseline correction to show raw enhancement (**A**, **B**), polynomial baseline correction followed by vector normalisation (**C**, **D**) or 1st order differentiation followed by vector normalisation (**E**, **F**).

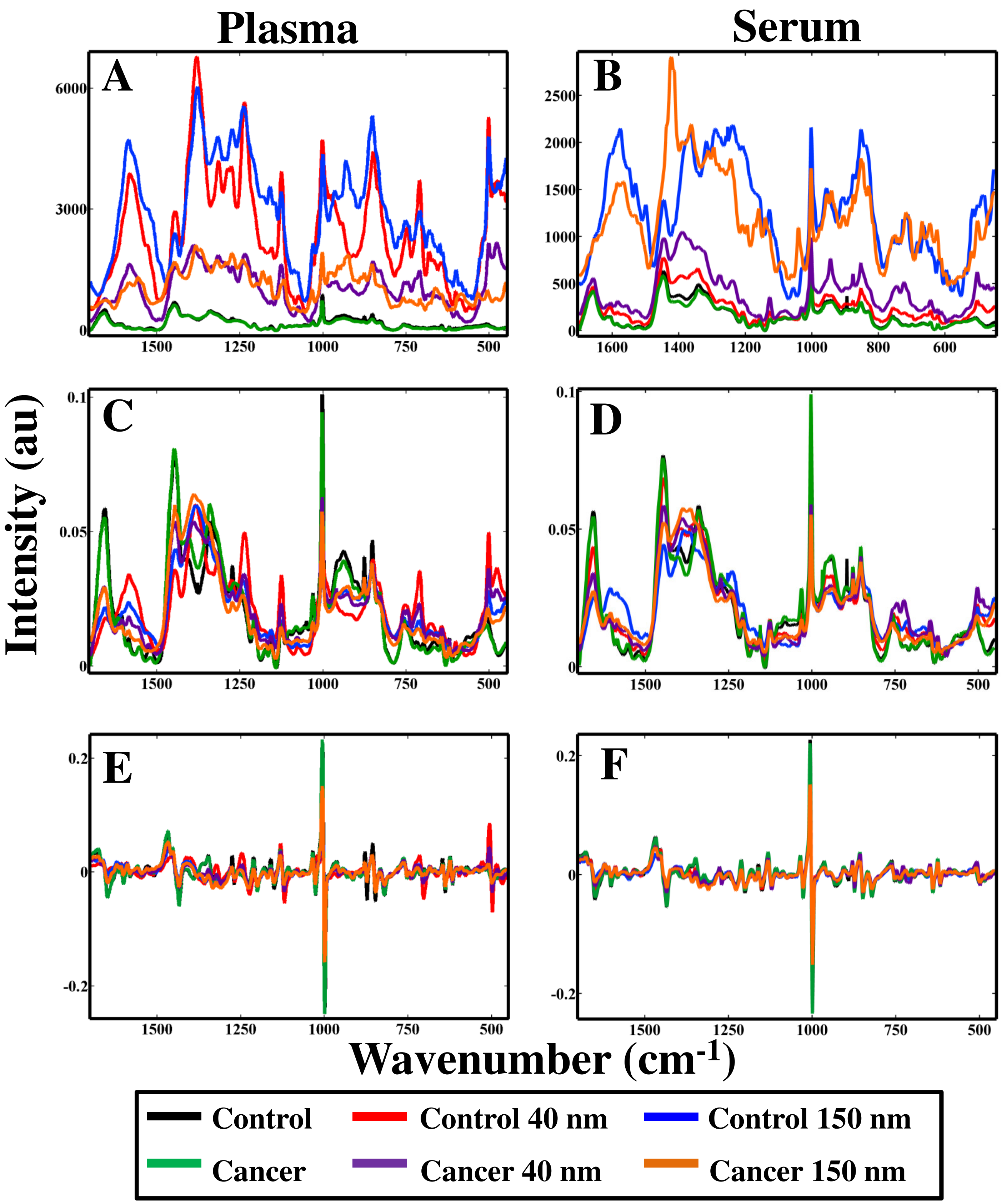

Figure S3. Influence of nanoparticles on SERS effect in blood plasma or serum samples. Class means spectra of all data acquired in this study, including non-enhanced spectra. Blood plasma (**A**, **C**, **E**) or serum samples (**B**, **D**, **F**) with or without gold nanoparticles: following polynomial baseline correction to show raw enhancement (**A**, **B**), polynomial baseline correction followed by vector normalisation (**C**, **D**) or 1st order differentiation followed by vector normalisation (**E**, **F**).
